# Anti‐influenza A (H1N1) virus effect of gallic acid through inhibition of virulent protein production and association with autophagy

**DOI:** 10.1002/fsn3.3852

**Published:** 2023-11-21

**Authors:** Cheng‐Chieh Chang, Huey‐Ling You, Huey‐Jen Su, I‐Ling Hung, Chao‐Wei Kao, Sheng‐Teng Huang

**Affiliations:** ^1^ Department of Chinese Medicine Kaohsiung Chang Gung Memorial Hospital and Chang Gung University College of Medicine Kaohsiung Taiwan; ^2^ Graduate Institute of Chinese Medicine China Medical University Taichung Taiwan; ^3^ Department of Laboratory Medicine Kaohsiung Chang Gung Memorial Hospital Kaohsiung Taiwan; ^4^ Department of Nursing Meiho University Neipu Shiang Taiwan; ^5^ Department of Chinese Medicine Jen‐Ai Hospital Taichung Taiwan; ^6^ Department of Chinese Medicine China Medical University Hospital Taichung Taiwan; ^7^ School of Chinese Medicine China Medical University Taichung Taiwan; ^8^ An‐Nan Hospital China Medical University Tainan Taiwan; ^9^ Cancer Research Center for Traditional Chinese Medicine, Department of Medical Research China Medical University Hospital Taichung Taiwan

**Keywords:** autophagy, gallic acid, influenza virus, LC3B, virulent protein

## Abstract

Influenza remains one of the most serious infectious diseases. Gallic acid is one of the most common and representative phenolic acids found in various plants. This is an interesting subject to explore how gallic acid could inhibit H1N1 influenza virus infection by reducing the production of virulent proteins and interrupting autophagy machinery for influenza virus replication on the host cell. Cellular viability was assessed by XTT assay. The inhibitory effects on the H1N1 influenza virus were assessed by hemagglutination assay, plaque assay, and qRT‐PCR. Western blot analysis was used for detecting protein levels of M1, M2, NP, LC3B, and beclin‐1. Autophagy activity was demonstrated by acridine orange staining assay. The result demonstrated that there was no cytotoxic effect of gallic acid on A549 cells, and gallic acid could restore the cellular viability of H1N1 influenza virus‐infected A549 cells within the experimental concentration treatment. Moreover, gallic acid could effectively restrain viral activity of the H1N1 influenza virus. After the treatment of gallic acid, the production of virulent H1N1 influenza virus proteins, that is, M1, M2, and NP protein were reduced. As for autophagic mechanism, both of the LC3B II conversion and the level ratio of LC3B II to LC3B I were notably decreased. The acridine orange staining assay also revealed decreased accumulation of autophagosomes in H1N1 influenza virus‐infected cells. In conclusion, gallic acid suppresses H1N1 influenza viral infectivity through restoration of autophagy pathway and inhibition of virulent M1, M2, and NP protein production.

## Graphical abstract

Gallic acid effectively suppressed the replication of H1N1 IAV with no cytotoxicity. Gallic acid apparently reduced the production of M1 and M2 proteins. Gallic acid could restore the autophagic mechanism induced by H1N1 IAV.

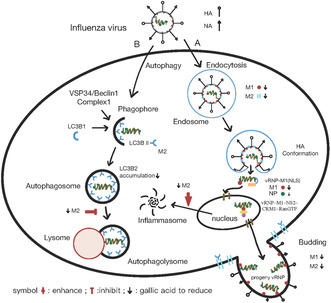

## 1 INTRODUCTION

Viral diseases still present severe challenges to human populations globally. Influenza remains a serious infectious disease, causing millions of severe cases and hundreds of thousands of deaths annually (Amarelle et al., 2017).

Four types of influenza virus are influenza A, B, C, and D. Influenza A virus (IAV) is the only influenza virus known to cause flu pandemics (Samji, 2009). There are 8 vRNPs retained in the IAV which encode transcripts for more than 10 essential viral proteins (PB2, PB1, PA, HA, NP, NA, M1, M2, NS1, and NS2). Of the viral proteins, HA is the most abundant viral envelope protein and is involved in viral entry (Dou et al., 2018). In addition, it triggers endocytosis of virus and induces fusion of the viral envelope with endosomal membrane to release vRNPs into host cell cytosol (Samji, 2009). M1 protein is the most abundant protein of influenza virus, forming a matrix layer to stabilize vRNPs inside the envelope (Bui et al., 2000). It also promotes the nuclear export process of progeny vRNPs and facilitates viral shedding of progeny virus (Huang et al., 2013; Rossman & Lamb, 2011). M2 protein, located at the viral envelope, works as a proton influx channel after endocytosis. The acidification of the viral core can break down the M1‐vRNP interaction, modify HA conformation, and allow fusion of endosome membrane with viral envelope to release vRNPs into host cell cytosol (Manzoor et al., 2017). During influenza viral shedding, M2 protein can facilitate the budding of progeny influenza virus from the host cell (Rossman & Lamb, 2011). Moreover, M2 protein is involved in induction of autophagy, and most importantly, interrupts the fusion of autophagosomes and lysosomes to escape the antiviral activity of host cells (Gannage et al., 2009; Zhang et al., 2014). It has also been demonstrated that the IAV can utilize M2 protein to relocalize LC3II protein to the cell surface membrane for budding of stable viruses (Manzoor et al., 2017). NP proteins form a major structural component of the vRNP complex. It also takes part in entry of vRNPs into host cell nucleus via nuclear localization signals (NLS) and export of vRNPs out of nucleus via nuclear export signals (NES) (Portela & Digard, 2002).

Autophagy is also an essential process for influenza viral replication. Autophagy is triggered hierarchically by a set of autophagy‐related gene proteins to initiate the formation of phagophores (Bello‐Perez et al., 2020; Portela & Digard, 2002). Upon viral infection, autophagy is induced to eliminate pathogenic viral components through autophagolysosome degradation. However, IAV can escape the degradation and utilize host autophagy for replication by blocking autophagosome–lysosome fusion (Zhang et al., 2014). It has been demonstrated that three viral proteins (i.e., M2, HA, and NS1 proteins) can induce host autophagy via AKT–mTOR‐dependent autophagy pathway (Zhang et al., 2014). During the formation of autophagosomes, the cytosolic light chain 3 (LC3B) protein is converted from soluble LC3B I to lipid bound LC3B II that is recruited to phagophore membrane. The quantification of autophagosomes can be assessed by the level of LC3B conversion (LC3B I conversion to LC3B II) (Quan & Lee, 2013). Therefore, autophagosomes accumulate in IAV‐infected host cells via inhibiting fusion of autophagosomes with lysosomes and autophagosomes degradation. Most importantly, IAV can exploit the autophagy machinery for the replication (Marino & Lopez‐Otin, 2004).

There is a common proverb that states that eating an apple a day keeps the doctor away. Gallic acid is one of the most important phenolic acids in many fruits, wines, tea, and herbal medicines (Del Rio et al., 2013). It possesses antioxidant effect, anti‐inflammatory response, and apoptosis‐inducing effect (Cheng et al., 2018; Dludla et al., 2018; Yang et al., 2018). Gallic acid and its derivatives have been used in ink dyes, tanning agents, and food additives for a long time (Badhani et al., 2015; Bardon et al., 2013). Gallic acid possesses various antiviral activities, including enterovirus 71, human rhinovirus, HSV‐2, and HCV (Choi, Song, Bhatt, & Baek, 2010; Choi, Song, Park, & Baek, 2010; Govea‐Salas et al., 2016; Kratz et al., 2008). The anti‐influenza virus potential of gallic acid has also been implied, but the precise underlying mechanisms remain unclear (Kim & Chung, 2018). Therefore, we herein aimed to explore the anti‐influenza effect of gallic acid on the alveolar epithelial cells and the underlying mechanisms.

## 2 MATERIALS AND MATERIALS

### 2.1 Virus preparation and cell culture

The pandemic influenza (H1N1) virus (A/California/07/2009) strains were used in this study. The study was performed in the Clinical Virology Laboratory of the Kaohsiung Chang Gung Memorial Hospital accredited by the College of American Pathologists. The human A549 cell line derived from NSCLC was obtained from American Type Culture Collection (ATCC). A549 cell line was cultured at 37°C in DMEM medium (Invitrogen Life Technologies), and 10% heated‐inactivated FBS, 250 ng/mL amphotericin B, and 100 𝜇g/mL penicillin and streptomycin were added to the cell culture media. H1N1 IAV at 1.17 multiplicity of infection was added into the monolayer of the A549 cells. After 1 h, the incubation solution was transferred and the cells were washed twice with phosphate‐buffered saline. Following the washing procedure, growth media containing experimental concentrations of gallic acid or 3‐methyladenine (3‐MA) was supplemented. 3‐MA was used as a positive control. Both the gallic acid and 3‐MA were purchased from Sigma‐Aldrich (Sigma‐Aldrich, USA). The cells were subsequently harvested for 72 h for further investigation.

### 2.2 Cytotoxicity assay

The XTT (tetrazolium hydroxide salt) assay was applied to detect the cell viability (Roche). Cells (1 × 10^5^ cells/well) were seeded in a 96‐well microplate and cultured for 72 h. Then, tetrazolium detection reagent was mixed in and incubated for 4 h. The absorbance was measured at 480 nm by a Sunrise microplate reader (TECAN) using 650 nm reference wavelength. Each experiment was executed in triplicate and performed at least thrice independently.

### 2.3 Hemagglutination assay

The supernatant of the cell culture was collected and 50 μL of supernatant was added into the round‐bottom 96‐well plate for each sample in duplicate. Fifty microliter of 0.5% human erythrocyte solution was incubated at 4°C for 3 h before agglutination was measured. The experiments were carried out independently in triplicate.

### 2.4 Plaque assay

Confluent monolayers of A549 cells in six‐well plates were infected with the H1N1 IAV (3 × 10^5^ PFU/mL) for 1 h. The media were then replaced with medium containing different concentrations of gallic acid (0, 0.5, 1, 5, and 10 μM) or 3‐MA (5 mM). The H1N1 IAV was isolated at 72 h after infection by freezing/thawing three times and centrifuging the supernatant at 500 × *g* for 10 min. Virus yields were measured via the plaque assay for influenza viruses in Madin–Darby kidney (MDCK) cells. Briefly, monolayer MDCK cells in six‐well plates (2 × 10^5^ cells/cm^2^) were infected with supernatants containing different concentrations of gallic acid (0, 0.5, 1, 5, 10 μM) or 3‐MA (5 mM)). Following absorption for 1 h, the MDCK cells were washed twice with prewarmed MEM medium and supplemented with immobilizing overlay gel (0.5% low melting agarose in minimal essential medium with 2 μg/mL trypsin). After addition of the overlay, the MDCK cells in six‐well plates were incubated for 72 h at 35°C with 5% CO_2_. The fixing procedure was using a 4% formaldehyde solution for 1 h. Running tap water was used to get rid of the agarose layer and the MDCK cells were stained by 1% (w/v) crystal violet. All assays were carried out in triplicate. The data were analyzed using Quantity One v.4.6.5 software from Bio‐Rad Laboratories.

### 2.5 Quantification of H1N1 IAV

The viral RNA was extracted using the LabTurbo automated system (Labturbo, Taiwan). Sixty microliterof elution was collected and then one‐step qRT‐PCRs was performed by 4X Fast Virus 1‐Step qRT‐PCR Probe MMIX (Topgen Biotech., TW) with 20X IAV qPCR assay (Topgen Biotech., TW) (primers/probe sequences are listed in Table S1). Standards for IAV qRT‐PCR were made by gene synthesis (Topgen Biotech., TW), and cloned into pGEM‐T vector (Promega). The qRT‐PCRs were implemented by the StepOnePlus™ Real‐Time PCR System (Thermo Fisher) with the following settings: 50°C for 15 min, 95°C for 30 s, followed by 45 cycles of 95°C for 10 s and 60°C for 30 s. Specimens were amplified three times with appropriate non‐template controls.

### 2.6 Western blotting analysis

Proteins were separated by the sodium dodecyl sulfate‐polyacrylamide gel (SDS‐PAGE) electrophoresis system. The concentration of proteins was detected by the Bradford method (Bio‐Rad). Purified sample proteins were separated by 10% SDS‐PAGE and transferred to polyvinylidene difluoride (Millipore) membrane. Immunoblot strips were reacted with the appropriate dilution of primary antibody: matrix protein 1/M1 and 2/M2 antibody (Santa Crus); *nucleoprotein* (*NP*) antibody (Invitrogen); microtubule‐associated proteins 1 light chain 3 B (LC3B) antibody (Novus) (16 Da for LC3B I; 14 kDa for LC3B II); beclin‐1 antibody (Novus); and anti‐glyceraldehyde 3‐phosphate dehydrogenase (GAPDH) (Merck) antibody at 25°C for 2 h. The blots were washed three times with Tris‐buffered saline containing Tween‐20 (TBST) and then incubated with 1:2000 dilution of horseradish peroxidase (HRP)‐conjugated secondary antibody (Jackson). Pierce enhanced chemiluminescent HRP substrate (Thermo Fisher) was used for immunodetection of target proteins. The measurements of the bands were analyzed using Quantity One software (Bio‐Rad).

### 2.7 Flow cytometry

Autophagic activity was measured by the acridine orange (AO) staining assay (Abcam). Cells were incubated with AO staining solution to measure the autophagic vacuoles in live cells. Briefly, the conditioned cells were incubated with AO staining solution diluted as 1:1000 at 37°C for 20 min. After washing, samples were analyzed with FACS Canto II (BD Biosciences) flow cytometer by a 488 nm excitation and a 525 nm band pass filter for FITC detection. The data were analyzed using FACS DIVA (BD Biosciences) software.

### 2.8 Statistical analyses

All statistical analyses were performed using the Statistical Product and Service Solutions (SPSS) v17.0 software (SPSS Inc.,). Results were represented as means ± standard deviation (SD). Statistical significance was determined by Student's *t*‐test. ANOVA was adopted on multiple experimental groups and one control group. A *p* value less than .05 is considered statistically significant. All experiments were repeated at least three times independently.

## 3 RESULTS

### 3.1 Gallic acid exhibited cytoprotection on A549 cells infected with the H1N1 IAV with no cytotoxicity

**FIGURE 1:**
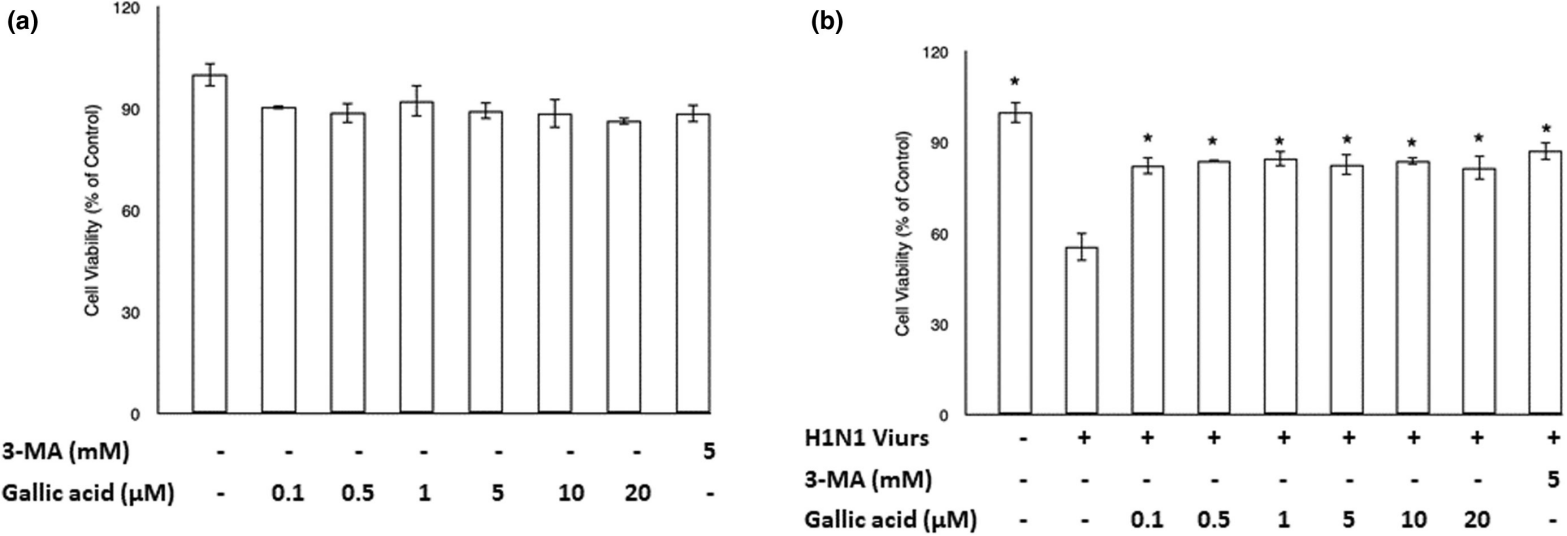
The cell viability of A549 cells with (a) or without (b) influenza A (H1N1) virus infection after treatment of gallic acid at different concentrations (0, 0.1, 0.5, 1, 5, 10, and 20 μM) and 3‐MA (5 mM) for 3 days. XTT assay was used to assess the cell viability. All data are expressed mean ± standard error of the mean in triplicate from three independent experiments. Asterisk (*) indicates *p* value smaller than .05 (*p* < .05) relative to the mean level in the viral control group.

XTT viability assay was applied to detect cytotoxic and cytoprotective effects of gallic acid on A549 cells. 3‐MA was adopted as a positive control reagent. Different concentrations of gallic acid (0, 0.1, 0.5, 1, 5, 10, and 20 μM) and 3‐MA (5 mM) were supplemented into A549 cell culture plates without (Figure 1a) or with (Figure 1b) H1N1 IAV infection for 1 h. The cell viability of A549 cells without H1N1 IAV infection was nearly intact after treatment with the experimental concentrations of gallic acid and 3‐MA for 72 h. Upon H1N1 IAV infection, less than 50% of A549 cells survived. After treatment with the experimental concentrations of gallic acid and 3‐MA for 72 h, the cell viability of A549 cells was restored up to 80%. The results indicated no cytotoxic effect of gallic acid below 20 μM on the A549 cells, while revealing a protective effect on H1N1 IAV‐infected A549 cells treated with gallic acid below 20 μM and 3‐MA at 5 mM for 72 h.

### 3.2 The inhibitory effect of gallic acid against H1N1 IAV infection

**FIGURE 2:**
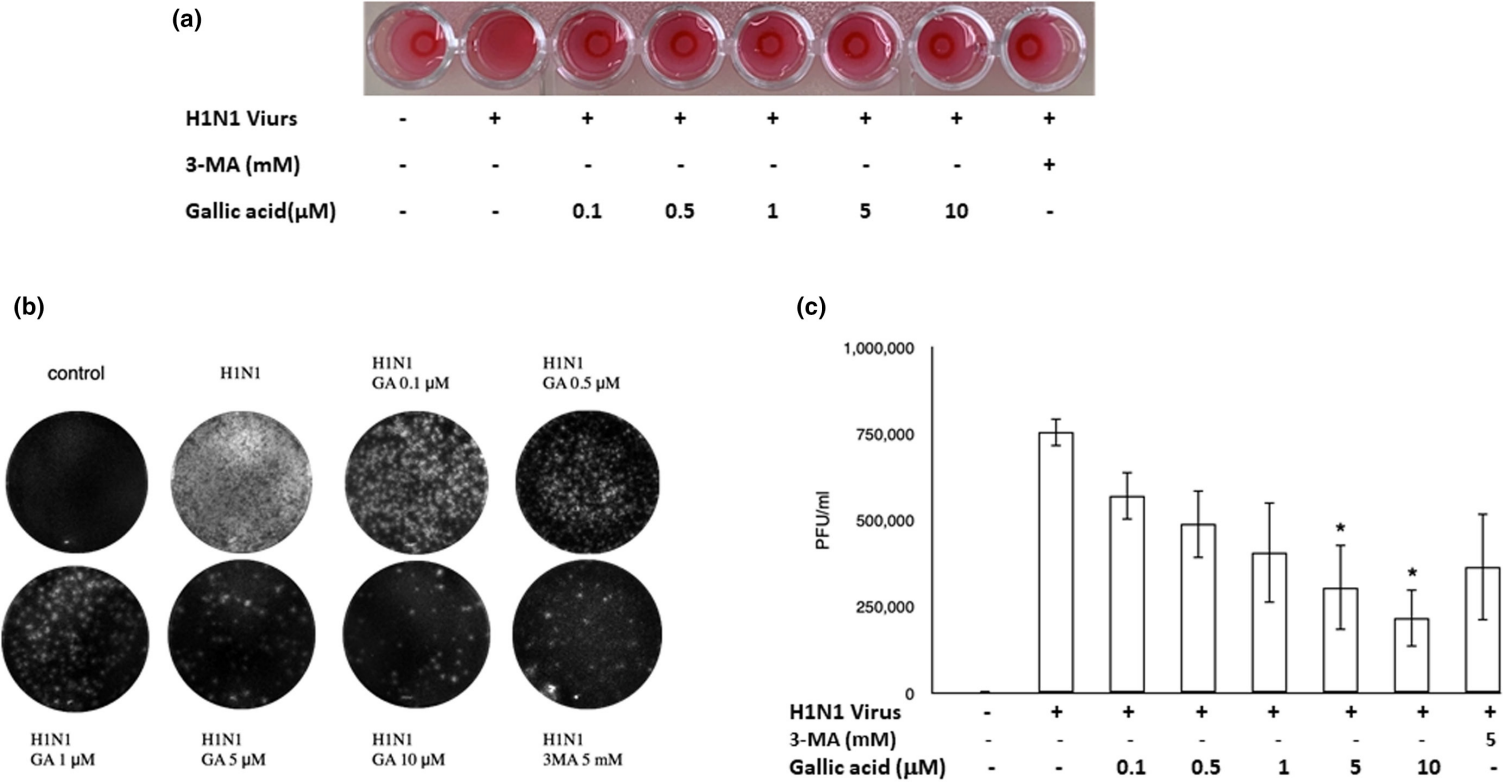
The inhibitory effect of gallic acid against influenza A (H1N1) virus infection was quantified by hemagglutination assay (a) and plaque assay (b). The titer of virus can be calculated in plaque‐forming units (PFU) per milliliter after treatment of gallic acid at different concentrations (0, 0.1, 0.5, 1, 5, and 10 μM) and 3‐MA (5 mM) (c). No H1N1 viral infection served as negative control and 3‐MA (5 mM) treatment as positive control. Data are presented by mean ± SEM. The results were undergone in duplicate three times independently. Asterisk (*) indicates *p* value smaller than .05 (*p* < .05) relative to the mean level in the viral control group.

Hemagglutination and plaque assays were performed to demonstrate the inhibitory effect of gallic acid against H1N1 IAV infection. A549 cells were treated with different concentrations of gallic acid (0, 0.1, 0.5, 1, 5, and 10 μM) and 3‐MA (5 mM) for 72 h, after which culture supernatants media were separated. The well without H1N1 IAV infection was regarded as a negative control, while the well with only H1N1 IAV infection was regarded as positive control. As the concentration of gallic acid increased, the hemagglutination assay of A549 cell supernatants changed from an agglutinated pattern to a non‐agglutinated button pattern (Figure 2a). The supernatant with 3‐MA (5 mM) treatment also exhibited a non‐agglutinated pattern. H1N1 IAV‐infected A549 cell culture supernatants were executed by plaque assays under the treatment of experimental concentrations of gallic acid (0, 0.1, 0.5, 1, 5, and 10 μM) (Figure 2b). Densitometric analysis of plaque formation quantified the inhibitory effect of gallic acid against H1N1 IAV infection in a dose‐dependent manner (Figure 2c). Both results of the hemagglutination and plaque assays indicated that both gallic acid and 3‐MA can prevent H1N1 IAV proliferation.

### 3.3 Gallic acid effectively decreased the viral load

**FIGURE 3:**
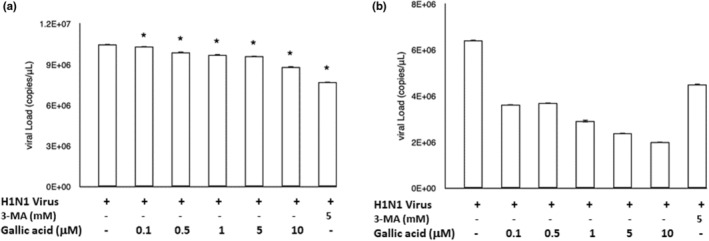
Relative quantification of influenza A (H1N1) virus accumulation in A549 cells (a) and the culture supernatant (b) by qRT‐PCR after treatment of gallic acid at different concentrations (0, 0.1, 0.5, 1, 5, and 10 μM) and 3‐MA (5 mM) for 72 h. Each experiment was executed in triplicate. The results are presented as the mean ± SEM from three independent experiments. Asterisk (*) indicates *p* value smaller than .05 (*p* < .05) relative to the mean level in the viral control group.

The qRT‐PCR was utilized to detect the quantification of virus nucleic acids in the A549 cells (Figure 3a) and in cell culture supernatants (Figure 3b). As the treatment concentrations of gallic acid increased (0, 0.1, 0.5, 1, 5, and 10 μM), viral load was decreased in both the H1N1 IAV‐infected A549 cells and the culture supernatants. The viral load decline was more notable in the culture supernatants than in the infected cells.

### 3.4 Gallic acid inhibited M1, M2, and NP protein production

**FIGURE 4:**
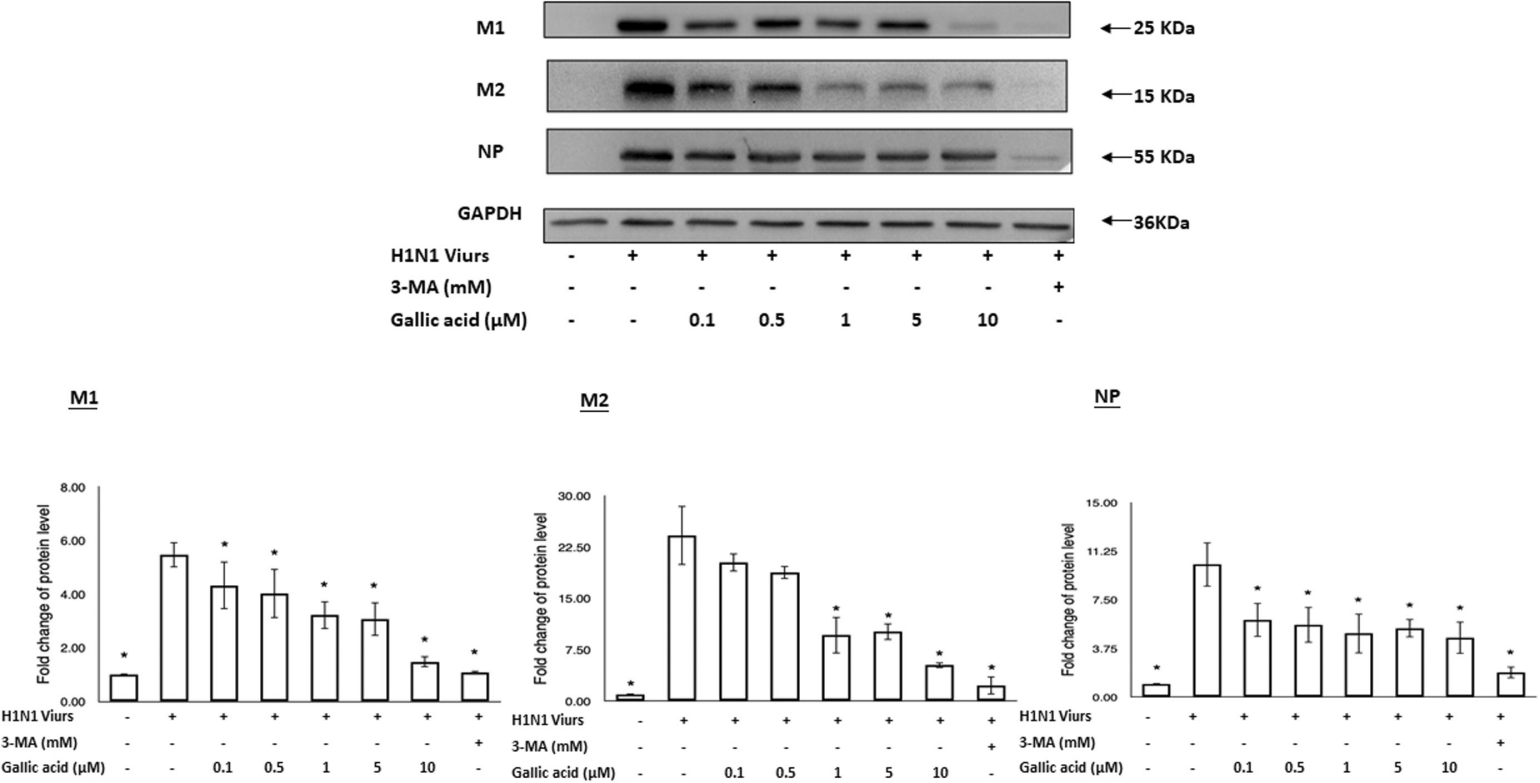
A549 cells were harvested at 72 h after influenza A (H1N1) virus infection, and equal amounts of protein from control and treated cell extracts were resolved on conventional 10% SDS‐PAGE. M1, M2, and NP proteins were analyzed by Western blot (a). The levels of M1, M2, and NP proteins were quantified by densitometric analysis (b). The results are represented as the mean ± SEM from three independent experiments. Asterisk (*) indicates *p* value smaller than .05 (*p* < .05) relative to the mean level in the viral control group.

A549 cells were treated with different concentrations of gallic acid (0, 0.1, 0.5, 1, 5, and 10 μM) for 72 h after H1N1 IAV infection. The amount of M1, M2, and NP proteins were analyzed by western blot (Figure 4a). As the treatment concentrations of gallic acid increased, the M1, M2, and NP proteins were reduced in the H1N1 IAV‐infected A549 cells (Figure 4b). The decrease in M2 protein was most notable, achieving a reduction of approximately 80% in a dose‐dependent manner. In addition, M1 protein reached a reduction of approximately 60%, while NP protein decreased by less than 50%. These results indicated that gallic acid could inhibit the virulent proteins (i.e., M1, M2, and NP proteins) production. The reduction in the M2 protein was the most prominent, following by that of the M1 protein.

### 3.5 Gallic acid can interfere with the autophagic machinery to inhibit the replication of H1N1 IAV

**FIGURE 5:**
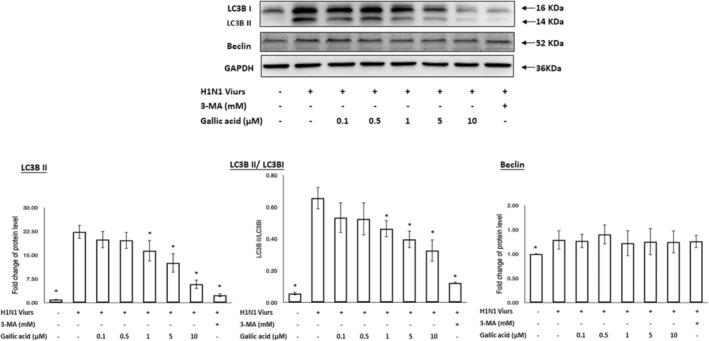
Western blot for LC3B and beclin‐1 on A549 cells after treatment of gallic acid at different concentrations (0, 0.1, 0.5, 1, 5, and 10 μM) and 3‐MA (5 mM) for 72 h (a). Quantification of LC3B and beclin‐1 from three independent experiments (b). The LC3B II‐to‐LC3B I ratio was also presented (c). Asterisk (*) indicates *p* value smaller than .05 (*p* < .05) relative to the mean level in the viral control group.

Both LC3B and beclin‐1 were designated as autophagic markers. Beclin‐1 is a primary regulator in the autophagy pathway. While LC3B is associated with the formation of phagosomes. Both LC3B II and beclin‐1 proteins were triggered after H1N1 IAV infection. After treatment with gallic acid, the amount of LC3B II protein notably decreased; however, there was no detectable change in the beclin‐1 protein. As a positive control, 3‐MA also inhibited LC3B II expression (Figure 5a,b). These results demonstrated that both gallic acid and 3‐MA could inhibit the accumulation of LC3B II. Moreover, the LC3B II‐to‐LC3B I ratio in this study also showed a downtrend under increasing concentration of gallic acid treatment (Figure 5c). Therefore, while the accumulation of LC3B II would enhance after H1N1 IAV infection, the accumulation of LC3B II could be reduced significantly after the treatment of gallic acid.

### 3.6 Gallic acid can reduce the accumulation of autophagosomes induced by H1N1 IAV infection

**FIGURE 6:**
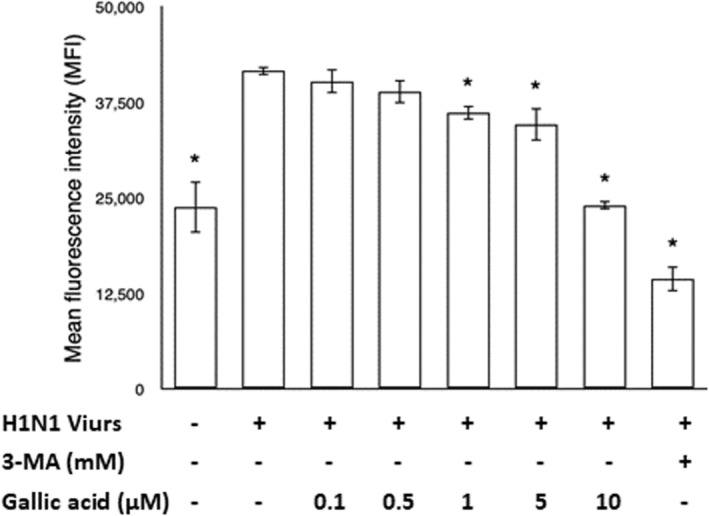
The detection of autophagy maturation was done by acridine orange (AO) staining assay. The assay was performed after treatment of gallic acid at different concentrations (0,0.1, 0.5, 1, 5, and 10 μM) and 3‐MA (5 mM). Data are presented as mean ± SEM. The results were undergone in duplicate from three independent experiments. Asterisk (*) indicates *p* value smaller than .05 (*p* < .05) relative to the mean level in the viral control group.

The maturation of the autophagy pathway was detected by AO staining assay. AO staining tags are fused in the N‐termini of the autophagosome marker LC3B. The change from autophagosome to autophagolysosome can be visualized by imaging the AO stain metachromatic shift from green to red (SenthilKumar et al., 2019). Autophagosomes accumulated on H1N1 IAV‐infected cells by hindering their fusion with lysosomes. Densitometric analysis of AO staining assay showed that the accumulation of autophagosomes gradually decreased as the treatment concentrations of gallic acid increased (Figure 6).

## 4 DISCUSSION

Although the antioxidant and anti‐inflammatory activities of gallic acid have been widely reported (Tanaka et al., 2018), we herein aimed to further clarify the anti‐H1N1 IAV mechanisms of gallic acid (You et al., 2018). We had demonstrated in the previous study that the 50% effective inhibition concentration (EC50) of gallic acid for H1N1 IAV was 2.6 mg/mL (15 mM), whereas the 50% cytotoxic concentration (CC50) of gallic acid was 22.1 mg/mL (129 mM) (You et al., 2018). It is more than six times concentration of gallic acid in this study. Therefore, we had selected the range of dose from 0.1 to 10, 20 micromolar in this study. There were lots of studies demonstrating the antioxidant, anti‐inflammation, and antiradiation effect of gallic acid at the concentration below 10 mM (Aborehab & Osama, 2019; Khorsandi et al., 2020; Tsai et al., 2018). We observed that gallic acid was a safe and non‐toxic compound for A549 cells at concentrations of up to 20 μM, and could rescue A549 cells from H1N1 IAV infection. The hemagglutination and MDCK plaque assays revealed that gallic acid could reduce the infectivity of H1N1 IAV. The inhibitory effect was demonstrated in a dose‐dependent manner. Therefore, gallic acid can effectively reduce the virus propagation of H1N1 IAV.

M1, M2, and NP viral proteins are essential virulent proteins in the IAV viral life cycle. This study demonstrated that gallic acid could most significantly reduce the production of M2 protein after IAV infection. M2 is mainly synthesized at the endoplasmic reticulum‐associated ribosomes; subsequently, it is translocated to the Golgi apparatus for viral assembly and budding (Dou et al., 2018). After IAV endocytosis, M2 can acidify endosomes and release the vRNPs into the cytosol for further replication (Cady et al., 2009). M2 protein can also protect HA fusion competence via alkalinizing the lumen of the Golgi apparatus to prevent HA premature conformational change (Alvarado‐Facundo et al., 2015). During late stage of infection, M2 protein can facilitate the release of the budding virus via clustering at the neck of the budding virus and resulting in membrane scission (Gannage et al., 2009). Meanwhile, M2 protein is required for autophagy viral replication by triggering initial stage of autophagy and inhibiting the fusion of autophagosome with lysosome (Zhirnov & Klenk, 2013). In addition, M2 protein can stimulate a detrimental inflammation effect in host cells via inflammasomes (Wang et al., 2019). Harnessing the crucial role of M2 protein can prevent the cytokine storm induced by influenza virus.

M1 protein works as a bridge between the lipid envelope of virus and vRNPs (Hilsch et al., 2014). Upon endocytosis, acidic environment of endosomes can not only induce fusion of virus envelope and endosomal membranes but also trigger a conformational change in M1 protein to release vRNPs into the cytosol (Dahmani et al., 2019). As the replication processes of influenza vRNPs primarily occur in the nucleus, the vRNP complex must be transported into the nucleus via nuclear localization signal on vRNPs (Eisfeld et al., 2015). After replication, various proteins help progeny vRNPs exit the nucleus. M1 protein is one of the most important proteins involved in vRNP nuclear export. It is performed by the formation of vRNP–M1–NS2–CRM1–RanGTP complex (Huang et al., 2013). Moreover, at the release stage of viral life cycle, M1 protein can facilitate viral shedding via interacting with transmembrane HA, NA, and M2 proteins, serving as a docking site for the recruitment of the viral RNPs and subsequently forming the interior structure of the emerging virus (Eisfeld et al., 2015). Therefore, gallic acid could fundamentally interfere with the entry and nuclear trafficking of vRNPs and viral shedding of IAV by significantly reducing the production of M1 protein.

Recent research has indicated that NP protein not only mediates efficient viral replication but also participates in the entry of vRNPs into nucleus through nuclear localization signal of NP protein and export of vRNPs from nucleus through nuclear export signals of NP protein (Turrell et al., 2013). Our experiments revealed that the reduction in the NP protein after treatment with gallic acid was not as notable as that of the M2 or M1 proteins.

Autophagy is also involved in the replication of H1N1 IAV. Upon IAV infection, autophagy is induced for H1N1 IAV replication. Both beclin‐1 and LC3B are important markers for autophagy.

Beclin‐1 has been linked to diverse biological processes. It can not only regulate diverse host cellular pathways induced by IAV infection, including apoptosis, endocytosis, and phagocytosis, but also initiate the formation of phagophores (Funderburk et al., 2010). In this study, there was no prominent change in beclin‐1 after treatment with experimental concentrations of gallic acid, which is consistent with previous studies (Law et al., 2014). H1N1 IAV infection can lead to the accumulation of LC3B II in the host cell via inhibiting autophagosome–lysosome fusion (Rossman & Lamb, 2009). In this study, the result also showed that LC3B II protein conversion was triggered and LC3B II protein accumulated after H1N1 IAV infection. After treatment with experimental concentration of gallic acid, the accumulation of LC3B II protein decreased notably. The results of the AO staining assay also demonstrated significant decrease in autophagosome accumulation after gallic acid treatment. Both results demonstrate that gallic acid can reduce the accumulation of autophagosomes induced by H1N1 IAV infection for viral replication. Although both the LC3B II and beclin‐1 proteins are triggered after H1N1 IAV infection, gallic acid presents significant effect on reduction in LC3B II but no obvious effect on beclin‐1. There are canonical and non‐canonical autophagy processes. Non‐canonical autophagy process does not require all of the ATG proteins to form autophagosome. Non‐canonical LC3 lipidation can reflect the extent of non‐canonical autophagy process (Liu et al., 2019). M2 protein is required for non‐canonical LC3 lipidation (Ulferts et al., 2021). In this study, gallic acid can inhibit the production of M2 production. Therefore, the reduction in LC3B II by gallic acid might partially be related to the inhibition of M2 production via non‐canonical LC3 lipidation. Whether the reduction in LC3B II by gallic acid was related to the canonical autophagy process is an intriguing subject for further study. Moreover, gallic acid can restore autophagy process via reduction in M2 protein which interrupts the fusion of autophagosomes and lysosomes.

According to the WHO report, the replication cycle of influenza viruses (from the time of entry to the production of new virus) is very quick, with shedding of the first influenza viruses from infected cells occurring after only 6 h. Therefore, we had executed more experiments to observe the protein synthesis inhibition for 6 h (even one cycle). The results showed that gallic acid possesses the inhibitive effect on viral protein synthesis for even one viral cycle (Figure S1). The synthesis of M2 protein is inhibited much more apparently than the synthesis of M1 and NP protein.

The change in LC3B II/ LC3B I during the first cycle of infection was examined (Figure S2). The amount of LC3B II/ LC3B I increased up to 4 times at 6 h post‐infection. The level of LC3B II/ LC3B I was also decreased after the treatment of gallic acid and 3 MA at 6 h post‐infection. Therefore, gallic acid could reduce the accumulation of autophagosomes induced by H1N1 IAV infection even early in viral infection, but due to short infection period, there was no dose‐dependent manner.

**FIGURE 7:**
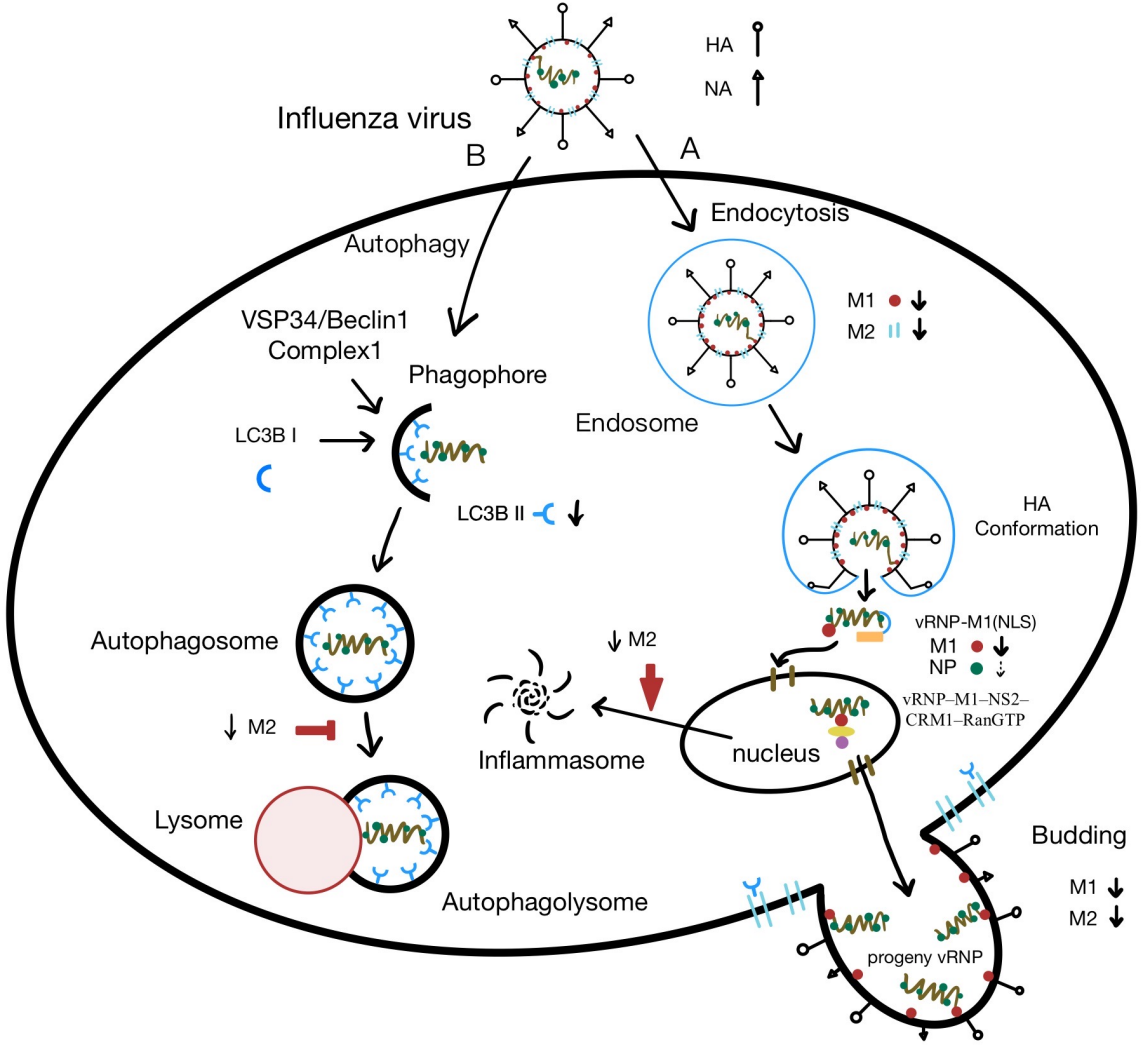
The inhibitory effect of gallic acid on H1N1 infection. A: After endocytosis, M2 can acidify endosomes and release the vRNPs into the cytosol. Then, vRNPs are transported into the nucleus via vRNP‐M1(NLS) binding. After replication, the formation of vRNP–M1–NS2–CRM1–RanGTP complex helps progeny vRNPs exit the nucleus. During the late stage of infection, M2 and M1 can facilitate budding of progeny virus. Moreover, M2 can enhance the production of inflammasome for the activation of inflammatory responses. Gallic acid can effectively decrease the production of virulent M1 and M2 production. B: For autophagy process, gallic acid has a significant effect on LC3B II reduction and can restore autophagy process via reduction in M2 protein which interrupts the fusion of autophagosomes and lysosomes.

In this study, we demonstrated that gallic acid could effectively inhibit the replication of H1N1 IAV on human lung A549 epithelium cells with no harm, apparently reducing the virulent virus proteins production of M1 and M2 and hindering autophagy viral replication by H1N1 IAV infection. A conclusive graph for the mechanisms of the inhibitory capacity of gallic acid on H1N1 infection was organized (Figure 7). Further studies of gallic acid in terms of the interplay among autophagy, apoptosis, and inflammation suppression are recommended.

## 5 CONCLUSION

Gallic acid can prevent H1N1 IAV infection through reducing virulent viral protein, for example, M2 and M1 proteins, and inhibiting the accumulation of autophagosomes induced by H1N1 IAV. Gallic acid interrupts the pathogenic viral life cycle of H1N1 IAV and reduces the infectivity of H1N1 IAV. Therefore, gallic acid can be offered as an adjuvant therapy for the treatment of influenza virus infection.

## AUTHOR CONTRIBUTIONS

**Cheng‐Chieh Chang:** Conceptualization (equal); formal analysis (equal); investigation (equal); project administration (equal); validation (equal); writing – original draft (equal). **Huey‐Ling You:** Data curation (equal); formal analysis (equal); investigation (equal); methodology (equal); resources (equal); software (equal); validation (equal). **Huey‐Jen Su:** Formal analysis (equal); investigation (equal); resources (equal); visualization (equal). **I‐Ling Hung:** Formal analysis (equal); investigation (equal); validation (equal); visualization (equal). **Chao‐Wei Kao:** Formal analysis (equal); investigation (equal); visualization (equal). **Sheng‐Teng Huang:** Conceptualization (equal); data curation (equal); funding acquisition (equal); methodology (equal); project administration (equal); supervision (equal); validation (equal); visualization (equal); writing – review and editing (equal).

## CONFLICT OF INTEREST STATEMENT

The authors declare no conflict of competing interests. Ethical Approval is not applicable to this article. This article does not contain any studies with human or animal subjects. There are no human subjects in this article and informed consent is not applicable.

## Supporting information


Figure S1.

Figure S2.

Table S1.

## Data Availability

The data that support the findings of this study are available from the corresponding author upon reasonable request.
