# Supplementary figures and images for: Anti‐influenza A (H1N1) virus effect of gallic acid through inhibition of virulent protein production and association with autophagy
Source: Food Sci Nutr. 2023 Nov 21;12(3):1605–15. doi: 10.1002/fsn3.3852 (PMC10916620; doi:10.1002/fsn3.3852)

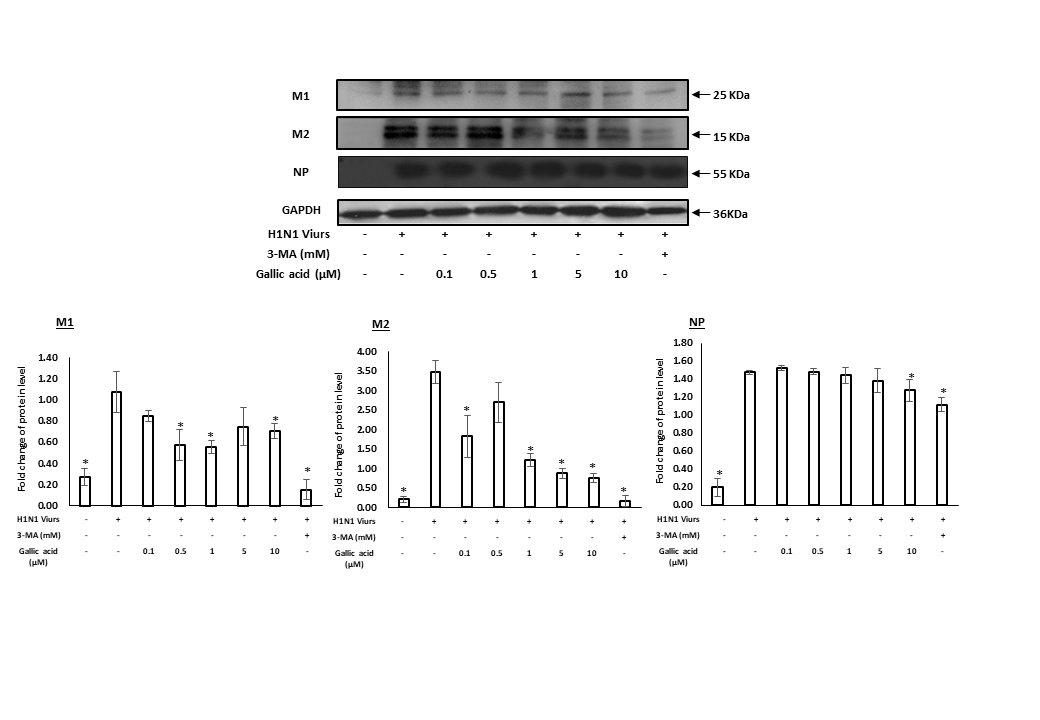

Supplement: Supplementary file 1 — Figure S1. [file FSN3-12-1605-s001.png]

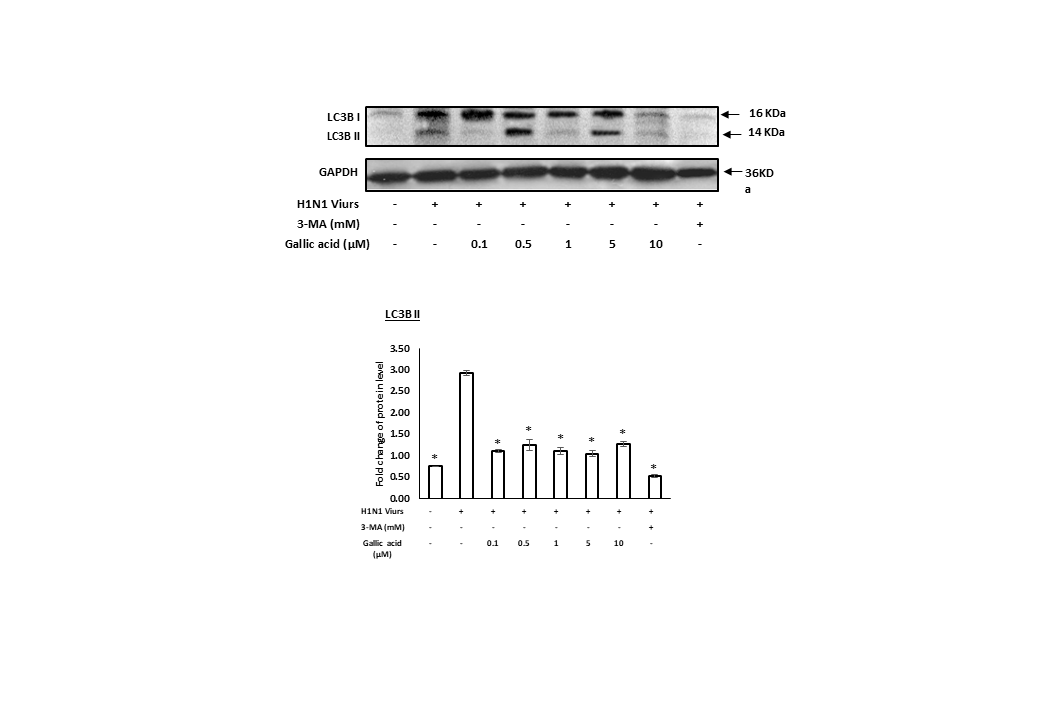

Supplement: Supplementary file 2 — Figure S2. [file FSN3-12-1605-s003.png]
