# Supplementary material for: Anti‐influenza A (H1N1) virus effect of gallic acid through inhibition of virulent protein production and association with autophagy
Source: Food Sci Nutr. 2023 Nov 21;12(3):1605–15. doi: 10.1002/fsn3.3852 (PMC10916620; doi:10.1002/fsn3.3852)
Supplement: Supplementary file 3 — Table S1. [file FSN3-12-1605-s002.docx]

**Supplementary Table 1**

**Oligonucleotides used for Influenza A (H1N1) virus amplification**

| Items | | Sequence (5'-3') | 5’labeled | 3’labeled | Species | Ref: Seq | Amplicon Length | Gene |
| --- | --- | --- | --- | --- | --- | --- | --- | --- |
| Primer | (1) Forward | GACCRATCCTGTCACCTCTGAC | ---- | ---- | Influenza A | CY085873.1 | 106bp | Matrix protein 1 |
|  | (2) Reverse | AGGGCATTYTGGACAAAKCGTCTA | ---- | ---- |  |  |  |  |
| Probe | | ACCGTGCCCAGTGAGCGAGGACT | FAM | BHQ1 |  |  |  |  |

FAM: 6-carboxyfluorescein ; BHQ1: Black Hole Quencher-1
